# Advances in the Treatment of Mycoses in Pediatric Patients

**DOI:** 10.3390/jof4040115

**Published:** 2018-10-11

**Authors:** Elias Iosifidis, Savvas Papachristou, Emmanuel Roilides

**Affiliations:** Infectious Diseases Unit, 3rd Department of Pediatrics, Faculty of Medicine, Aristotle University School of Health Sciences, Konstantinoupoleos 49, 54642 Thessaloniki, Greece; iosifidish@gmail.com (E.I.); savvas.gpap@gmail.com (S.P.)

**Keywords:** invasive fungal infections, neonates, antifungal agents, children

## Abstract

The main indications for antifungal drug administration in pediatrics are reviewed as well as an update of the data of antifungal agents and antifungal policies performed. Specifically, antifungal therapy in three main areas is updated as follows: (a) Prophylaxis of premature neonates against invasive candidiasis; (b) management of candidemia and meningoencephalitis in neonates; and (c) prophylaxis, empiric therapy, and targeted antifungal therapy in children with primary or secondary immunodeficiencies. Fluconazole remains the most frequent antifungal prophylactic agent given to high-risk neonates and children. However, the emergence of fluconazole resistance, particularly in non-albicans Candida species, should be considered during preventive or empiric therapy. In very-low birth-weight neonates, although fluconazole is used as antifungal prophylaxis in neonatal intensive care units (NICU’s) with relatively high incidence of invasive candidiasis (IC), its role is under continuous debate. Amphotericin B, primarily in its liposomal formulation, remains the mainstay of therapy for treating neonatal and pediatric yeast and mold infections. Voriconazole is indicated for mold infections except for mucormycosis in children >2 years. Newer triazoles-such as posaconazole and isavuconazole-as well as echinocandins, are either licensed or under study for first-line or salvage therapy, whereas combination therapy is kept for refractory cases.

## 1. Introduction

Invasive fungal infections (IFIs) are important causes of excessive morbidity and mortality in pediatrics. *Candida* spp. and *Aspergillus* spp. are the most common fungi causing IFIs in neonates, infants, children, and adolescents. *Mucorales, Cryptococcus neoformans,* and other fungi are less frequent causes [1]. The incidence of IFIs in various countries and hospitals depends on many factors; however, the degree of exposure to fungal elements and the predisposition of the host to develop IFI are the two main determinants of the fungal epidemiology in particular patient populations.

There are various factors that can increase the risk for developing an IFI in pediatrics. Premature birth and very-low-birth weight (VLBW) have been recognized as an important factor for increased IFIs, especially for invasive candidiasis (IC) in neonatal age. For older infants and children, primary or especially secondary immunodeficiencies are main underlying conditions predisposing to IFIs. Secondary immunodeficiencies are those resulted from cancer-related chemotherapy, stem cell or organ transplantation, and administration of various immunosuppressive agents. Other risk factors are the existence of foreign bodies, and most frequently central catheters predisposing to biofilm development, and overuse of broad-spectrum antibiotics, resulting in antibiotic-induced modified microbiota.

Specific characteristics of neonates and young children are extremely relevant to antifungal drug therapy—such as pharmacokinetics, drug metabolism, age-dependent adverse effects, route of drug administration, and limited clinical data. The impact of these pediatric factors has been recognized as a major issue during development of pediatric guidelines of IFI management [2].

In this article, the main indications for antifungal drug administration in pediatrics will be reviewed as well as an update of the data of antifungal agents as well as antifungal policies will be performed. Specifically, antifungal therapy in three main areas will be updated as follows:

(a) Prophylaxis of premature neonates against invasive candidiasis;
(b) management of candidemia and meningoencephalitis in neonates; and
(c) prophylaxis, empiric therapy, and targeted antifungal therapy in children with primary or secondary immunodeficiencies.

Doses and indications of antifungal agents in pediatrics are summarized in Table 1.

## 2. Prophylaxis of Preterm Babies against Candidiasis

Measures to prevent colonization of infants, especially of VLBW neonates, by *Candida* spp. are the cornerstone of prevention of IFIs among them [3,62,63]. Avoidance of horizontal transmission by rigorous infection control measures, rational use of broad-spectrum antibacterial agents (especially third-generation cephalosporins and carbapenems), and proper handling of central venous catheters appear to be extremely important [3,64].

Early administration of probiotics is associated with prevention of *Candida* colonization in neonates; however, their efficacy in reducing IFIs and mortality, their safety, risk-benefit potential, optimal dosage, and duration of administration is still unclear [57,58,59,60]. A subsequent meta-analysis did not show any significant impact of probiotic administration in reducing incidence of late onset sepsis [61]. Thus, the impact of probiotics in reducing IFIs in neonates needs further study.

Several neonatal units around the world use fluconazole as means of IFI prevention depending on their IFI incidence [65,66,67,68]. A more recent network meta-analysis, including 11 randomized controlled studies of fluconazole prophylaxis, confirmed that fluconazole at 3, 4, or 6 mg/kg twice weekly was superior to placebo preventing invasive candidiasis (IC) and IC-related mortality in preterm neonates, but failed to find any difference between these three dosage regimens [17]. Although the study could not demonstrate any effect on long-term toxicity or the development of resistance due to the design of the studies, it recommended the smallest dose of 3 mg/kg twice weekly as sufficient for prophylaxis as well as being better tolerated, minimizing exposure, and reducing cost. Ericson et al. collected patient-level data from four US-based randomized studies and conducted an aggregated analysis of these data. They found that fluconazole prophylaxis significantly reduced IC and *Candida* colonization in preterm infants, whereas it had no impact on the fluconazole resistance rate during the period of the study [69]. Nevertheless, neonatal intensive care units (NICUs) should avoid overuse of prophylactic fluconazole and try to use infection control bundles and antibiotic stewardship to avoid increased use of fluconazole with subsequent increases of *Candida* minimal inhibitory concentrations to fluconazole. Until more definitive results on drug exposure exist, fluconazole 3–6 mg/kg twice weekly for 42 days is recommended for <1000 gr preterm neonates in NICUs with a relatively high incidence of IC (>10%) [4]. The rational of this twice weekly regimen is to reduce drug exposure (leading to adverse effects, especially long-term neurodevelopmental consequences) as well as the development of resistant fungal isolates showing, nevertheless, sufficient efficacy [17]. In addition, one study found that the twice-weekly regimen at a dose of 3 mg/kg twice weekly was not significantly associated with neurodevelopmental sequelae after 8–10 years or impaired quality of life [70]. As the long-term complications of neonatal period are frequently multifactorial, further studies are needed to identify the impact of antifungal prophylaxis.

Nystatin has been also proposed as an alternative antifungal prophylactic regimen in neonates, because it is safe, well tolerated, effective, and cheap, although data are limited and especially for neonates weighing less than 750 g. It may be used in an alternative antifungal prophylaxis setting with fluconazole shortages or resistance [4]. In a non-inferiority randomized study, prophylactic use of nystatin or fluconazole reduced both the incidence of colonisation and IFIs in VLBW neonates [71]. Of interest, in another study of fluconazole or nystatin oral prophylactic use in VLBW infants (birth weight <1500 g), the nystatin group showed an unexplained proportion of deaths [72]. The role of oral non-absorbable antifungals to reduce *Candida* gut colonization and indirect risk of IFIs in the neonates has not been adequately studied [73].

## 3. Management of Invasive Candidiasis in Neonates

Significant differences in IFI pathophysiology and antifungal drug pharmacokinetics between premature neonates and older patients emphasize the need to further evaluate the effectiveness and safety of different antifungal drugs and dosing in the neonatal period [74]. In a recent multicenter population-based study of antifungal treatment in premature neonates in Germany, 5.4% of VLBW and 10.7% of extremely-low-birth weight (ELBW) neonates received any empirical or targeted antifungal therapy. Among them, 95.5% of them received empirical instead of targeted therapy. Fluconazole was the most frequently used agent followed by liposomal amphotericin B and caspofungin [75].

Prompt initiation of empiric antifungal treatment (before knowing the positive culture), especially in high-risk neonates, is a prerequisite to efficiently combat candidemia, leading to increased survival without neurodevelopmental impairment [76,77]. The empiric treatment should be based on epidemiology of the specific NICU; any premature infant with clinical or microbiological evidence of IC should be treated as having disseminated disease and hematogenous *Candida* meningoencephalitis (HCME) [3]. In all neonates with suspected IC, a lumbar puncture and a retinal examination should be performed and in case of persistent positive blood cultures, ultrasonography of head, heart, and kidneys for end-organ dissemination is strongly recommended [4]. Removal of a central venous catheter, if possible, is very important for IC treatment to diminish the niche of a persistent *Candida* biofilm [4]. No consensus on the duration of antifungal therapy in the neonatal period has been established; however, most NICUs treat IC for a minimum of 14 days after negative cultures [4]. The duration of therapy for end-organ infections—such as endocarditis and renal infection—is even less clear, but a duration of six weeks or longer is recommended [78].

Non-culture assays have been studied for the early diagnosis of IC in neonates and thus initiation of preemptive therapy with the goal to improve the outcome. (1,3)-β-d-glucan (BDG), a reliable biomarker of most clinically relevant fungi in adults, has been studied very little in neonates with variable results [79,80]. In a more recently published study of premature neonates comparing those with proven or probable invasive yeast infections with controls, BDG was shown to have promising results [81]. The authors suggested a higher threshold, i.e., 106 or 125 pg/mL, may be used, but interpretation of BDG results in neonates should be made with caution, taking into consideration all limitations. A multicenter study evaluated the role of polymerase chain reaction (PCR) and BDG in the early diagnosis of IC in premature neonates in comparison to blood culture [82]. With a cutoff value of either >80 pg/mL or >120 pg/mL used, the authors found that *Candida* PCR and serum BDG are useful as complementary diagnostic techniques to detect IC in premature neonates.

T2 magnetic resonance assay is a very promising assay that may give the result of positivity and *Candida* spp. identification much faster (three to four hours) than culture (which may take up to four to five days) [83]. The use of valid diagnostics with good sensitivity and specificity may make the treatment more target-oriented and much earlier. No published data of T2 magnetic resonance assay exist with IC in neonates.

Deoxycholate amphotericin B (DAMB) continues to be useful in the treatment of IFIs in NICU. While its pharmacokinetics in neonates are considerably variable [84], the dosage regimen recommended is 1 mg/kg/d [3,4,5]. However, its use is limited by its adverse effects of nephrotoxicity and hypokalemia [5]. Therefore, frequent monitoring of electrolytes and renal function is recommended during therapy.

Pharmacokinetic data are available on the liposomal and lipid complex preparations of amphotericin B and they support their use in neonates; however, the optimal dosage and duration of therapy is difficult to establish [16]. Amphotericin B lipid formulations are administered in increased daily dosages (up to 10-fold), and are characterized by high tissue concentrations in macrophage-rich organs (lungs, liver, spleen), a decrease in infusion-related adverse effects, and a marked decrease in renal toxicity. Dosing of liposomal amphotericin B (LAMB) in neonatal IC ranges from 2.5–7 or 3–5 mg/kg/d according to ESCMID or IDSA guidelines, respectively [3,4]. When comparing the efficacy and safety of DAMB and lipid amphotericin B (AMB) formulations (liposomal and lipid complex) in neonatal IFIs, there are no significant differences noticed, although the number of studies is limited [85,86,87]. While there is no specific clinical information for the optimal regimen for HCME, LAMB penetrates the central nervous system (CNS) in a preclinical model of HCME and has antifungal activity in the brain [8,9].

Fluconazole has potent in vitro activity against almost all *Candida* spp., fair pharmacokinetic properties with good penetration to the CNS, and has been used in neonates with IC at 12 mg/kg/d [3,4]. A loading dose of 25 mg/kg during the first day is probably needed to achieve steady state levels within 24 h. Fluconazole resistance has emerged, particularly in non-*C. albicans* species, reaching high levels in some regions and local epidemiology should be considered [3]. Nevertheless, in Europe and in America, resistance of main *Candida* spp. causing IC in neonates (*C. albicans* and *C. parapsilosis*) remains low (<5%) [88,89,90,91,92]. Fluconazole should not be used as empiric therapy in neonates that have prophylactically received fluconazole in the past.

Micafungin is the only echinocandin approved for use in infants aged less than three months in the EU and Japan, but not in the USA [40]. An analysis of nine clinical trials containing neonates showed that micafungin has a substantial efficacy, with a success rate of IC treatment reaching 73% in both premature and non-premature groups [41] that is somewhat less than the success rate found in adults (approximately 83%) [42]. Because of increased clearance in neonates, an increased dose of 4–10 mg/kg/d is recommended [43]. The most appropriate doses to achieve levels in the brain parenchyma are 7–10 mg/kg/d. This has been suggested from pharmacokinetics and pharmacodynamics studies of micafungin in experimental HCME [44]. A population pharmacokinetics study of micafungin in neonates and young infants showed that a higher weight-based dose is required for infants than for adults to achieve effective CNS drug concentrations [45]. This has been validated in a recent clinical trial in neonates, and with the use of a Monte Carlo simulation it was shown that micafungin was efficient and well tolerated at a dose of 10 mg/kg and that there was no need for a loading dose (some authors have used 15 mg/kg) [46]. In a recently published very small study of micafungin at 10 mg/kg/d (20 patients) vs. deoxycholate amphotericin B at 1 mg/kg/d (10 patients) in infants up to four months of age with IC, there was no difference in fungal-free survival and both agents were well tolerated by the infants [93].

As neonatal IC is frequently followed by HCME, higher doses of echinocandins are required to achieve adequate drug levels in cerebrospinal fluid (CSF). In a study of 18 neonates with IC, three of whom also had meningitis, micafungin was given at a high dose of 8–15 mg/kg/d. Micafungin concentrations achieved in the CSF were 0.80–1.80 mg/L. While 78% of the subjects treated had clinical resolution of IC, five had neurologic impairments. In three patients treated with 10–15 mg/kg/d, marked γ-GT elevations were observed that were improved after dose reduction [94]. Micafungin has been used for shunt lock therapy combined with systemic treatment to treat shunt-associated *Candida* CNS infections [47].

Micafungin has few drug-drug interactions and an acceptable safety profile, but transaminase monitoring is recommended during treatment. While echinocandins have a favorable safety profile, the lack of clinical study data inhibits recommendations as first-line agents in neonates [93,95].

Caspofungin, despite being the first echinocandin used in adults, has limited data for neonates and infants less than three months [51,52]. In a clinical trial, a dosage of 25 mg/m^2^ given once daily was well tolerated and reached the same pharmacokinetic/pharmacodynamic (PK/PD) parameters as in adults treated with the recommended dose of 50 mg [53]. In a case report of device-associated meningitis in a premature neonate, the use of the above recommended dose (25 mg/m^2^) resulted in adequate caspofungin concentrations in the CSF and a microbiological and clinical response without the need for device removal [96].

Anidulafungin is not approved for use in neonates and infants less than three months of age. A systematic review of anidulafungin showed no drug-related adverse events, and good pharmacodynamics [97,98]. The efficacy of anidulafungin in neonatal HCME was evaluated using a well-established and proven rabbit model and applying a mathematical model to translate the results to humans. It was concluded that the current dosing regimen of 1.5 mg/kg/d with a loading dose of 3 mg/kg is not sufficient to treat HCME [97]. Another study exploring the safety and pharmacokinetics of anidulafungin in neonates and young infants showed that administration of anidulafungin at 1.5 mg/kg/d caused exposure levels that were similar to those of children on the same dose and adults that received the recommended dose of 100 mg/d [99].

## 4. Prophylaxis, Empiric Therapy, and Targeted Antifungal Therapy in Children with Primary or Secondary Immunodeficiencies

### 4.1. Prophylaxis

Prevention of IFIs in high-risk immunocompromised patients is very important. An appropriate bundle of preventive strategies consists of environmental strategies to lower the chance of fungal acquisition and primary/secondary antifungal drug prophylactic administration. Implementation of high efficiency-particulate air filters, room cleaning, and decontamination, protective clothing, care regarding food, and rational use of antibiotics with an active antibiotic stewardship program require special consideration and are prerequisite preventive measures in high-risk children.

Data on antifungal prophylaxis in children are limited and recommendations are mainly extrapolated from adults. On the other hand, it should be kept in mind that pediatric patients differ from adults in underlying conditions and physiology, in pharmacologic considerations, including dosing, toxicity, and administration, and have a much smaller evidence base, upon which recommendations are relied. Evidence-based recommendations for antifungal prophylaxis have been developed for the following categories of secondary immunodeficiencies: Acute myeloid leukemia, high-risk acute lymphatic leukemia, recurrent acute leukemia, intensive care unit (ICU) admission, and the presence of graft-versus-host disease (GVHD) after allogeneic hematopoietic stem cell transplantation (HSCT) [10]. In addition, patients with primary immunodeficiencies may need life-long antifungal prophylaxis, the kind of which may depend on the predominant fungi causing IFIs in the particular immunodeficiency.

According to ESCMID and to ECIL’s four guidelines, antifungal prophylaxis should be administered until engraftment after HSCT [3,10]. Antifungal prophylaxis should be continued after engraftment in case of GVHD (with antifungal agents active against both yeasts and molds). In children with HSCT, but without GVHD, prolongation of antifungal therapy after engraftment may be considered. In this case, the duration of antifungal prophylaxis should be determined by the immune recovery and the end of immunosuppression [10].

The most frequent antifungal agent prophylactically administered to high-risk pediatric patients is fluconazole, based on trials that have shown that patients with allogeneic HSCT receiving fluconazole presented a significant better long-term follow-up probably due to less severe gut GVHD [18,19,20,21]. Fluconazole is safe and well tolerated up to 12 mg/kg/d and doses up to 10 mg/kg/d were successfully used in a prophylaxis trial [22,23,24]. The pediatric fluconazole dose recommended for prophylaxis ranges between 6 and 12 mg/kg/d depending on the patient’s age and weight [18,23].

Since fluconazole does not possess anti-mold activity, studies have compared other antifungal agents with fluconazole to further reduce the incidence of mold infections in high-risk children. In a meta-analysis, antifungal prophylaxis with fluconazole was compared to a mold-active azole, or amphotericin B or echinocandin in adult and pediatric cancer patients receiving chemotherapy or HSCT, but no difference in overall mortality between the two arms was observed (RR 1.0; 95% CI 0.88–1.13) [100]. However, mold active agents significantly reduced both IFI episodes and IFI-related deaths, but with the cost of a higher prevalence of adverse events [100]. Other mold-active azoles were evaluated in high-risk patients for their preventive activity against mold infections. In particular, itraconazole reduced the incidence of IFIs, but due to its adverse effects and frequent drug-drug interactions it is not favorite to the clinicians as prophylaxis in high-risk pediatric patients [29,30]. In general, azoles are associated with many drug-drug interactions, leading to changes in concentrations of azoles or of interacting medications [101]. The list of drugs that have been implicated is long, including, but not limited to, rifampin, phenytoin, carbamazepine, vinca alkaloids, and calcineurin inhibitors [101].

Voriconazole is a broad-spectrum azole with anti-mold activity, but its use in pediatrics is complicated by inadequate drug exposure and by significant drug-drug interactions [102,103]. A large randomized controlled trial (RCT) has compared voriconazole to fluconazole in adult and pediatric patients with allogeneic HSCT [31]. The results have shown no difference in fungal-free or overall survival at 180 days. The proposed maintenance dose is 8 mg/kg twice daily for intravenous administration and 9 mg/kg twice daily for oral administration for all children less than 12 years of age, and for those 12–14 years of age weighing <50 kg [10,31]. In a recent nationwide, multicenter observational study regarding antifungal prophylaxis in children with cancer, the use of voriconazole was safe and well tolerated [104]. Therapeutic drug monitoring should be applied to optimize its serum levels between 1–1.5 and 5 mg/L [10,105]. It is not recommended for patients <2 years [10].

Posaconazole is another alternative choice for hematological/oncological patients, including those with GVHD. Factors limiting its use in children include variable plasma concentrations in pediatric patients, especially those less than 13 years of age, lack of an intravenous formulation (since these children typically have nausea, mucositis, and poor oral intake during the at-risk period), and unreliable absorption of oral suspension. One of the first clinical trials was specifically conducted in patients with GVHD [106]. This double-blind trial compared fluconazole to posaconazole in patients 13 years and older with acute Grade II–IV or chronic extensive GVHD. Patients receiving posaconazole prophylaxis had lower rates of proven and probable IFIs and a lower fungal-related mortality. Currently, posaconazole is recommended for use in patients >13 years of age with HSCT.

Posaconazole was approved for antifungal prophylaxis (for *Aspergillus* and *Candida* infections) in patients with acute myeloid leukemia, myelodysplastic syndromes, GVHD, or in patients undergoing HSCT, in whom a long neutropenic period due to chemotherapy is expected [12,13,35]. Accumulated data from studies in children, including all ages, with neutropenia have shown that posaconazole is safe and efficacious when used for prophylaxis in these patients [39,107]. In comparison with other azoles (voriconazole and itraconazole), posaconazole was well-tolerated, safe, and effective oral antifungal prophylaxis in pediatric patients who underwent high-dose chemotherapy and HSCT [107,108,109]. Recently, the use of a new posaconazole formulation (gastro-resistant tablets) has been studied in HSCT pediatric patients for antifungal prophylaxis [38]. In this study, the use of posaconazole as a suspension or tablet had similar efficacy, but patients who received a posaconazole tablet achieved much earlier the targeted plasma concentration than those who received the suspension formulation [38]. Posaconazole has also been used in patients with primary immunodeficiencies, including chronic granulomatous disease (CGD) [36]. Although a daily dose of 120 mg/m^2^ body surface given in three doses has been proposed for oral posaconazole suspension [37], the optimal dosage needs to be further studied in children, including the new formulation [39]. For children ≥13 years, a dose of 300 mg per day in one dose of gastro-resistant tablets (preferred formulation) is recommended. Alternatively, 600 mg per day of oral suspension in three divided doses for prophylaxis (not approved in the EU in patients <18 years). The oral solution administration needs therapeutic drug monitoring (TDM).

Data on echinocandin prophylaxis in pediatrics are limited, with only one study revealing that a higher proportion of patients receiving micafungin than fluconazole had no proven/probable/possible IFI at four weeks following HSCT [48]. There was no difference in rates of proven or probable IFI or overall and fungal-related mortality. However, the number of pediatric subjects enrolled was small (n = 84) and a reduction in the incidence of proven or probable IFI was not demonstrated. Micafungin was also tested as antifungal prophylaxis at 2 mg/kg/d in pediatric patients with allogeneic HSCT [49]. The main disadvantages for widespread echinocandin use are a lack of oral formulation and cost.

Caspofungin has been shown to be at least equivalent to itraconazole as antifungal prophylaxis with little caspofungin-related adverse events [54]. Two retrospective studies conducted in children undergoing HSCT caspofungin was safe and with a similar efficacy with a comparator antifungal agent, which was liposomal amphotericin B in one study and micafungain in the other [55,56].

Patients with primary immunodeficiencies being at high risk for IFIs or presenting as chronic fungal infections (i.e., chronic mucocutaneous candidiasis, CGD, severe combined immunodeficiency, and others) may require life-long antifungal prophylaxis [25,110,111]. Fluconazole for anti-*Candida* prophylaxis or maintenance therapy and a mold-active azole—such as itraconazole, voriconazole, or posaconazole—for anti-*Aspergillus* prophylaxis are appropriate and are thoroughly reviewed elsewhere [25,26,27,28].

### 4.2. Empiric Therapy

Empiric fever-driven antifungal therapy has been considered as standard of care in hemato-oncological patients at high risk for invasive fungal disease with neutropenia, who present with refractory or a new fever of at least four days, despite broad-spectrum antibacterial therapy [11]. Both ECIL’s four guidelines in 2014 and a recent update of clinical practice guidelines in children with cancer and HSC recipients recommend the following options in pediatric patients of all age groups: LAMB (1–3 mg/kg/d) or caspofungin (loading dose 70 mg/m^2^/d, followed by 50 mg/m^2^/d) [10,11]. Children with febrile and neutropenia with low risk for IFI have no benefit with starting empiric antifungal treatment. Nowadays, a more diagnostics-driven empiric (or pre-emptive) antifungal therapy is desirable based on findings of biomarkers, including galactomannan, and BDG, as well as imaging of chest and other organs [11,112]. This eliminates the need for administration of empiric antifungal therapy in neutropenic patients, who present with fever of non-fungal etiology. In a very recent randomized, multicenter clinical trial conducted in Chile, pre-emptive antifungal therapy was non-inferior to standard empiric driven antifungal therapy in pediatric patients aged less than 18 years with cancer and high-risk febrile neutropenia [113]. In addition, pre-emptive therapy was associated with statistically significant less antifungal exposure.

A randomized, double-blinded, multicenter study compared caspofungin and LAMB for empiric antifungal therapy in pediatric patients with persistent febrile neutropenia (FN). It revealed that caspofungin (50 mg/m^2^/d) and LAMB (3 mg/kg/d) were comparable in safety, tolerability, and efficacy [114]. Caspofungin doses can be increased to 70 mg/m^2^/d if clinically indicated (maximum dose of 70 mg/d). Another multicenter study evaluated the efficacy and safety of micafungin at a median dose of 3.0 mg/kg/d and a duration of 13.5 days for FN in pediatric patients with hematological malignancies, and revealed that micafungin may be an alternative safe and effective agent to treat pediatric patients with FN [50].

Empirical therapy in adult ICU patients has been shown to be of no benefit when using a fever criterion [3,4]. Similarly, a recent meta-analysis in non-neutropenic pediatric and adult ICU patients found that non-targeted antifungal therapy (including prophylactic, pre-emptive, and empiric antifungal treatment) compared to placebo or no therapy had no significant reduction in overall mortality [115]. Diagnostic-driven empiric antifungal therapy has been developed both for children and adults, but with significant limitations [116]. Currently, no conclusive evidence exists for the use of galactomannan, BDG, mannan antigen/antibody, and fungal PCR in children with high or low risk for IFD [116,117]. Improvement of current biomarkers alone or in combination [118] is warranted. A new and promising biomarker, T2 magnetic resonance assay, can detect the five most common isolated *Candida* species within a few hours in whole blood [119]. This T2 magnetic resonance biomarker has already been applied in pediatric children with and without candidemia and although there were few patients, a high accuracy was found [83].

In general, patients with a refractory or new fever episode in the pediatric intensive care unit (PICU), despite broad-spectrum empirical antibacterial therapy, who are at high risk for *Candida* infection with moderate-to-severe disease, hemodynamic instability recent azole exposure, or at high risk for *C. glabrata* or *C. krusei* infections require empiric treatment with an echinocandin or LAMB [4,12].

### 4.3. Targeted Therapy

LAMB at 3–5 mg/kg/d has been approved as a first-line treatment of IFIs in pediatrics, including invasive aspergillosis (IA) and IC [3,4,10]. Amphotericin B lipid complex at 5 mg/kg/d can be also used [13]. Other indications for AMB administration in children include blastomycosis, coccidioidomycosis, histoplasmosis, and endemic mycoses, whereas LAMB at a higher dose (≥5 mg/kg) is the first-line treatment of mucormycosis [13,14]. The combination of LAMB with flucytosine is proposed to treat difficult fungal infections, such as *Candida* meningitis and endocarditis [15]. For the management of cryptococcosis in children, data are limited. The main principle is to start induction therapy for meningoencephalitis using LAMB combined with flucytosine for at least two weeks, followed by fluconazole for a long period [6]. According to 2010 guidelines, the proposed dose regimen in children for CNS and disseminated cryptococcal disease is DAMB (1 mg/kg/d iv) in combination with flucytosine (100 mg/kg/d orally in four divided doses) for two weeks, followed by fluconazole (10–12 mg/kg/d orally) for eight weeks; for AmB-intolerant patients, either liposomal AmB (5 mg/kg per day) or amphotericin B lipid complex (ABLC) (5 mg/kg per day). Fluconazole is also proposed as a maintenance treatment at a dose of 6 mg/kg per day orally, while for cryptococcal pneumonia, fluconazole use is recommended (6–12 mg/ kg) for 6–12 months [6,7].

Among echinocandins, caspofungin is most commonly used to treat IFIs in pediatric patients [120]. An open-label prospective study of caspofungin for the treatment of IC and IA in patients aged three months to 17 years reported success (defined as complete or partial response) at the end of therapy in 5/10 (50%) patients with IA and 30/48 (62.5%) patients with IC without presenting any major adverse effects during treatment [121]. Another prospective study evaluated caspofungin in 83 patients with IA, most of which were refractory to other therapies. An overall favorable response to caspofungin was noted in 45% of patients [122]. A recent systematic review and meta-analysis of three randomized controlled trials of a high quality, but with small sample size, in children and neonates found a favorable outcome for patients treated with caspofungin in comparison to amphotericin B [123]. Based on these randomized trials as well as observational studies, caspofungin was approved for pediatric patients as second-line therapy of IA and as primary targeted therapy of IC in Europe [3,10].

Micafungin pediatric data are also limited. A retrospective study of patients <2 years of age found nine clinical studies having enrolled patients of this age [41]. Treatment-related adverse events were recorded in 23% of patients, with no difference between prematurely and non-prematurely born infants. For a subgroup of 30 patients with IC, treatment success was achieved in 73% in both premature and non-premature groups. The safety and pharmacokinetics of micafungin at 3 mg/kg and 4.5 mg/kg once-daily were evaluated in children with proven, probable, or suspected IC [124]; both dosages of micafungin were well tolerated. In a randomized double-blind trial of micafungin (2 mg/kg/d) versus LAMB (3 mg/kg/d) in 98 children aged <16 years, both drugs achieved similar success rates for treating candidemia/IC [125]. In a recent meta-analysis, the use of micafungin for the prevention or treatment of IFIs in neutropenic cancer pediatric patients had significantly more success rates than comparators, but similar mortality rates [126]. According to ESCMID and ECIL’s four guidelines, micafungin is licensed for targeted therapy of IC at a dose of 2–4 mg/kg [3,10].

A meta-analysis was recently conducted to evaluate the limited clinical data of echinocandins vs. other antifungal therapy for safety and efficacy in the treatment of IC in children [127]. In particular, from four randomized clinical trials (324 patients), two confirmed IC (micafungin vs. LAMB and caspofungin vs. LAMB) and two empirical therapy trials (caspofungin vs. DAMB and caspofungin vs. LAMB) were included. There was no significant difference between echinocandins and the comparator in terms of treatment success (OR = 1.61, 95% CI 0.74–3.50) and incidence of treatment-related adverse events (OR = 0.70, 95% CI 0.39–1.26). However, fewer children treated with echinocandins discontinued treatment due to adverse events than amphotericin B formulations (OR = 0.26, 95% CI 0.08–0.82, *p* = 0.02).

Pediatric data on anidulafungin efficacy and safety are restricted in only one published pediatric study to date [128]. Anidulafungin was well tolerated. Pediatric patients receiving 0.75 mg/kg/d or 1.5 mg/kg/d had anidulafungin concentration profiles similar to those of adult patients receiving 50 or 100 mg/d, respectively. In a prospective study conducted in a single center in Argentina, a total of 55 patients received anidulafungin for treatment or prophylaxis due to a temporary shortage of amphotericin B [129]. The majority of the children had bone marrow transplantation and anidulafungin was well tolerated and efficacious when given at a loading dose of 3 mg/kg/d and a maintenance dose of 1.5 mg/kg/d [129]. Preliminary data of an open study of anidulafungin to patients two years to 17 years were recently presented, showing that anidulafungin at doses of 3 mg/kg as a loading dose followed by 1.5 mg/kg/d was effective, with a global response success rate of 70.8% at end-of-iv treatment and an acceptable tolerability and safety profile in children diagnosed with IC [130]. Similar data are currently being analyzed for patients one month to two years of age. ESCMID/ECIL/IDSA guidelines recommend a dose of 3 mg/kg as a loading dose followed by 1.5 mg/kg/d [3,4,10]. Anidulafungin is not licensed yet for patients younger than 18 years.

Fluconazole can be safely used in IC caused by fluconazole-susceptible organisms in pediatric patients of all ages who are in a stable condition and have not received prior azole therapy [3,10,12]. Fluconazole or echinocandin can also be used as an initial therapy for candidemia in non-neutropenic patients, depending on the disease severity and possibility of azole resistance. According to IDSA guidelines, an echinocandin should be started for patients with moderately severe to severe illness or for patients with recent azole exposure, while fluconazole is recommended for patients with a less critical condition and without recent azole exposure or risk for a fluconazole resistant candida [4].

Voriconazole is indicated in pediatric patients as first line therapy for IA as well as for scedosporiosis, fusariosis, and other IFIs in pediatric patients, who are intolerant of or refractory to conventional antifungal therapy [10,33,34]. The recommended voriconazole dose for the treatment of IA is based on the age and weight of the child and TDM is strongly recommended [10]. As voriconazole has linear PK and high variability in children 2–11 years old, the dose for children is a loading of 9 mg/kg/dose × 2 followed by maintenance of 8 mg/kg/dose × 2 with pos 9 mg/kg/dose × 2. Higher voriconazole dosages or even given every eight hours have been suggested for children and especially in younger children aged less than two years of age [32].

Mucormycosis is a life-threatening IFI particularly affecting children with hematological malignancies [131]. Posaconazole is active against mucormycosis, and when combined with other antifungal drugs, can be effective in immunocompromised children and be used as a salvage therapy [132,133,134]. According to ECIL’s four recommendations, posaconazole can be used in children >13 years for scedosporiosis and fusariosis and as second line treatment for mucormycosis [10]. In a multicenter, phase three, open-label study in juvenile (age range 8–17 years old) and adult (18–64 years) patients who were intolerant of or had IFIs refractory to standard antifungal therapies, posaconazole at 800 mg/d as an oral suspension in divided doses was administered to almost all patients [135]. The overall success rates and adverse event profiles were comparable. Posaconazole plasma concentrations were similar for juvenile and adult patients, suggesting that clinical outcomes are expected to be similar in adults and children with refractory IFIs. Nowadays, the gastro-resistant tablets of posaconazole have eliminated the need for TDM. For the treatment of children ≥13 years, a dose of 300 mg per day in one dose (day 1, two doses of 300 mg) of gastro-resistant tablets (preferred formulation) is recommended. Alternatively, a dose of 800 mg per day in two or four divided doses of oral solution is recommended (not approved in the EU in patients <18 years). The oral solution administration needs therapeutic drug monitoring (TDM).

There are limited data for the use of posaconazole for the treatment of IFIs in children aged less than 13 years of age. In a recent study, 13 pediatric cancer patients were treated with posaconazole at a median dose of 12.5–16.5 mg/kg/d and 77% of them achieved the targeted concentration of 1 μg/mL within the first week [136]. Similarly, a retrospective analysis of the administration of oral posaconazole suspension for the treatment of fungal infections was conducted in 12 patients aged less than 13 years [36]. In this study, which included mostly male patients with CGD, posaconazole dose ranged from 18.5–47.9 mg/kg/d, the daily frequency varied from two to four doses, and plasma concentrations ranged from 0.23–2.16 μg/mL. TDM is the same in children and adults, 0.7 for prophylaxis and 1 mg/L for treatment.

Isavuconazole, a new triazole with activity against both *Aspergillus* and *Mucorales,* and both oral and intravenous formulation, has been recently approved for use in adults above 18 years [137]. Currently, its pharmacokinetics are being studied in children from 1–18 years for the intravenous regimen and from 6–18 years for the oral regimen (ClinicalTrials.gov NCT03241550). It has been used for treating a few children with mucormycosis successfully [138].

A number of rarer IFIs may be encountered in immunocompromised children—such as invasive fusariosis, scedosporiosis, or trichosporonosis. A review of these infections in children has been published [139]. Antifungal therapy for them in infants or children does not differ from that in adults even though some of the active agents (such as voriconazole) cannot be given in young patients. Surgical intervention and adjunctive immunotherapy have been used in some cases [140].

## 5. Conclusions

Fluconazole remains the most frequent antifungal prophylactic agent given to high-risk neonates and children. However, the emergence of fluconazole resistance, particularly in non-*albicans Candida* species, should be considered during preventive or empiric therapy. In VLBW neonates, although fluconazole is used as antifungal prophylaxis in NICU’s with a relatively high incidence of IC, its role is under continuous debate. The mainstay of therapy for treating neonatal and pediatric yeast and mold infections remains amphotericin B, primarily in its liposomal formulation. Voriconazole is indicated for mold infections except mucormycosis in children >2 years. Newer triazoles—such as posaconazole and isavuconazole—and echinocandins are either licensed or under study for first-line or salvage therapy, whereas combination therapy is kept for refractory cases.

## Figures and Tables

**Table 1:** Antifungal drugs in pediatric patients.

Drug	Route	Dosage	Indications for Use	References
**AMB formulations**				
DAMB	IV	• 1 mg/kg/d	• Treatment of IFIs in NICU	[3,4,5]
			• CNS and disseminated cryptococcal disease (combined with 5FC)	[6,7]
LAMB	IV	• 2.5–7 or 3–5 mg/kg/d	• Treatment of IC in neonates	[3,4]
		• No dosage established	• Treatment of HCME in neonates	[8,9]
		• 1–3 mg/kg/d	• Empiric fever-driven therapy in hemato-oncological patients at high risk for invasive fungal disease with neutropenia and refractory or new fever of at least 4 days, despite broad-spectrum antibacterial therapy	[10,11]
		• 3 mg/kg/d	• Empiric treatment in patients with refractory or new fever episode in the PICU, despite broad-spectrum empirical antibacterial therapy, who are at high risk for Candida infection with moderate-to-severe disease, hemodynamic instability, recent azole exposure, or at high risk for C. glabrata or C. krusei infections	[4,12]
		• 3–5 mg/kg/d	• First-line treatment of IFIs (IA, IC) in pediatrics	[3,4,10]
		• ≥5 mg/kg	• First-line treatment of mucormycosis	[13,14]
		• 5 mg/kg/d	• *Candida* meningitis and endocarditis (combined with 5FC)	[15]
		• 5 mg/kg/d	• Second-line treatment of CNS and disseminated cryptococcal disease (AMB-intolerant patients)	[6,7]
ABLC	IV	• No dosage established	• Treatment of IC in neonates	[16]
		• 5 mg/kg/d	• Treatment of IFIs (IA, IC) in pediatrics	[13]
		• 5 mg/kg/d	• Treatment for: Blastomycosis, coccidioidomycosis, histoplasmosis, endemic mycoses	[13,14]
		• 5 mg/kg/d	• Second-line treatment of CNS and disseminated cryptococcal disease (AMB-intolerant patients)	[6,7]
**Azoles**				
FLC	IV, PO	• 3, 4, or 6 mg/kg twice weekly	• Prophylaxis against IC in <1000 gr preterm neonates in NICUs with incidence of IC >10%	[4,17]
	IV	• 25 mg/kg loading dose, followed by 12 mg/kg/d	• Treatment of IC in neonates (according to local epidemiology)	[3,4]
	PO	• 6–12 mg/kg/d	• Antifungal prophylaxis in high-risk, immunocompromised pediatric patients (not active against molds)	[18,19,20,21,22,23,24]
		• 6-12 mg/kg/d	• Anti-*Candida* prophylaxis in patients with primary immunodeficiencies at high risk for IFIs or presenting as chronic fungal infections	[25,26,27,28]
	IV, PO	• 10–12 mg/kg/d	• Maintenance treatment of CNS and disseminated cryptococcal disease	[6,7]
		• 6–12 mg/kg	• Treatment of Cryptococcal pneumonia	[6,7]
		• 12 mg/kg/d	• Treatment of IC provided that: It is caused by fluconazole-susceptible organisms, the patient is in stable condition, and has not received prior azole therapy	[3,10,12]
ITC	PO	• 200 mg b.i.d.	• Antifungal prophylaxis in high-risk, immunocompromised pediatric patients (anti-mold activity)	[29,30]
		• 200 mg b.i.d.	• Anti-*Aspergillus* prophylaxis in patients with primary immunodeficiencies at high risk for IFIs or presenting as chronic fungal infections	[25,26,27,28]
VRC	IV PO	• 8 mg/kg b.i.d. • 9 mg/kg b.i.d.	• Antifungal prophylaxis in pediatric patients with allogeneic HSCT (anti-mold activity)	[10,31]
	IV, PO	• Same as above	• Anti-*Aspergillus* prophylaxis in patients with primary immunodeficiencies at high risk for IFIs or presenting as chronic fungal infections	[25,26,27,28]
	IV	• Children 2-11 years: loading 9 mg/kg/dose x 2, followed by maintenance 8 mg/kg/dose x2, with pos 9 mg/kg/dose x2 Children <2 years: higher voriconazole dosages or doses given every 8 h	• First-line therapy for IA	[10,32]
	IV	• Same as above	• Treatment for: Scedosporiosis, fusariosis (cases of intolerance of or refractoriness to conventional antifungal therapy)	[10,33,34]
PSC	PO (susp.)	• 600 mg/d, (given in 3 doses)	• Antifungal prophylaxis for hematological/oncological patients with acute myeloid leukemia, myelodysplastic syndromes, GVHD or in patients undergoing HSCT, in whom a long neutropenic period due to chemotherapy is expected	[12,13,35]
		• 600 mg/d, (given in 3 doses)	• Antifungal prophylaxis in primary immunodeficiencies (including CGD)	[36,37]
		• 800 mg/d in 2–4 divided doses	• Treatment for: Scedosporiosis, fusariosis	[10]
			• Second line treatment for mucormycosis	[10]
	PO (tabl.)	• Children ≥13 years old: 300 mg/d, q.d. Children <13 years: dose not established	• Antifungal prophylaxis in HSCT pediatric patients	[38]
			• Antifungal prophylaxis in primary immunodeficiencies (including CGD)	[39]
			• Treatment for: Scedosporiosis, fusariosis • Second-line treatment for mucormycosis	[10]
ISA	IV	• No dosage established	• PK is being studied in age group 1–18 years	[ClinicalTrials. gov NCT03241550]
	PO	• No dosage established	• PK is being studied in age group 6–18 years	
**Echinocandins**				
MFG	IV	• 4–10 mg/kg/d	• Second-line treatment of IC in neonates	[40,41,42,43]
		• 7–10 mg/kg/d	• Second-line treatment of HCME in neonates	[44,45,46]
	Lock Therapy		• Shunt lock therapy in shunt-associated *Candida* CNS infections (combined with systemic treatment)	[47]
	IV	• 2 mg/kg/d	• Antifungal prophylaxis in allogeneic HSCT pediatric patients	[48,49]
		• 3 mg/kg/d (median dose)	• Alternative treatment choice in pediatric patients with FN	[50]
		• 2–4 mg/kg	• Targeted therapy of IC	[3,10]
CAS	IV	• 25 mg/m^2^, q.d.	• Treatment of IC in neonates and infants <3 months (limited data)	[51,52,53]
		• 50 mg/m^2^/d	• Antifungal prophylaxis in HSCT pediatric patients	[54,55,56]
		• 70 mg/m^2^/d loading dose, followed by 50 mg/m^2^/d	• Empiric fever-driven therapy in hemato-oncological patients at high risk for invasive fungal disease with neutropenia and refractory or new fever of at least 4 days, despite broad-spectrum antibacterial therapy	[10,11]
		• Same as above	• Second-line therapy of IA • Primary targeted therapy of IC (in Europe)	[3,10]
AFG	IV	• 3 mg/kg loading dose, followed by 1.5 mg/kg/d	• Not yet licensed for patients <18 years	[3,4,10]
**Others**				
Nystatin	PO	• 100,000 u (1 mL)	• Second-line choice for antifungal prophylaxis in neonates, in cases of: Fluconazole shortages or resistance	[4]
Probiotics	PO	• No dosage established	• Prevention of *Candida* colonization in neonates (unclear efficacy)	[57,58,59,60,61]

AMB, Amphotericin B; DAMB, Deoxycholate amphotericin B; IFI, Invasive fungal infection; NICU, Neonatal intensive care unit; 5FC, flucytosine; LAMB, Liposomal amphotericin B; IC, Invasive candidiasis; HCME, Hematogenous *Candida* meningoencephalitis; PICU, Pediatric intensive care unit; IA, Invasive aspergillosis; ABLC, AMB lipid complex; FLC, fluconazole; ITC, itraconazole; VRC, voriconazole; HSCT, *Hematopoietic stem cell transplantation;* PSC, posaconazole; susp., suspension; GVHD, Graft-versus-host disease; tabl., tablets; CGD, chronic granulomatous disease; ISA, Isavuconazole; MFG, micafungin; FN, Febrile neutropenia; CAS, caspofungin; AFG, anidulafungin; q.d., once a day; b.i.d., twice a day.

## References

[1] Pana Z.D., Roilides E., Warris A., Groll A.H., Zaoutis T. (2017). Epidemiology of Invasive Fungal Disease in Children. J. Pediatr. Infect. Dis. Soc..

[2] Pana Z.D., Kougia V., Roilides E. (2015). Therapeutic strategies for invasive fungal infections in neonatal and pediatric patients: An update. Expert Opin. Pharmacother..

[3] Hope W.W., Castagnola E., Groll A.H., Roilides E., Akova M., Arendrup M.C., Arikan-Akdagli S., Bassetti M., Bille J., Cornely O.A. (2012). ESCMID* guideline for the diagnosis and management of Candida diseases 2012: Prevention and management of invasive infections in neonates and children caused by *Candida* spp.. Clin. Microbiol. Infect..

[4] Pappas P.G., Kauffman C.A., Andes D.R., Clancy C.J., Marr K.A., Ostrosky-Zeichner L., Reboli A.C., Schuster M.G., Vazquez J.A., Walsh T.J. (2016). Clinical Practice Guideline for the Management of Candidiasis: 2016 Update by the Infectious Diseases Society of America. Clin. Infect. Dis..

[5] Turkova A., Roilides E., Scharland M. (2011). Amphotericin B in Neonates: Deoxycholate or Lipid Formulation—What is the Right Choice?. Curr. Opin. Infect. Dis..

[6] Perfect J.R., Dismukes W.E., Dromer F., Goldman D.L., Graybill J.R., Hamill R.J., Harrison T.S., Larsen R.A., Lortholary O., Nguyen M.H. (2010). Clinical practice guidelines for the management of cryptococcal disease: 2010 update by the infectious diseases society of America. Clin. Infect. Dis..

[7] Perfect J.R., Bicanic T. (2015). Cryptococcosis diagnosis and treatment: What do we know now. Fungal Genet. Biol..

[8] Scarcella A., Pasquariello M.B., Giugliano B., Vendemmia M., de Lucia A. (1998). Liposomal amphotericin B treatment for neonatal fungal infections. Pediatr. Infect. Dis. J..

[9] Silver C., Rostas S. (2018). Comprehensive drug utilization review in neonates: Liposomal amphotericin B. J. Pharm. Pharmacol..

[10] Groll A.H., Castagnola E., Cesaro S., Dalle J.H., Engelhard D., Hope W., Roilides E., Styczynski J., Warris A., Lehrnbecher T. (2014). Fourth European Conference on Infections in Leukaemia (ECIL-4): Guidelines for diagnosis, prevention, and treatment of invasive fungal diseases in paediatric patients with cancer or allogeneic haemopoietic stem-cell transplantation. Lancet Oncol..

[11] Lehrnbecher T., Robinson P., Fisher B., Alexander S., Ammann R.A., Beauchemin M., Carlesse F., Groll A.H., Haeusler G.M., Santolaya M. (2017). Guideline for the Management of Fever and Neutropenia in Children With Cancer and Hematopoietic Stem-Cell Transplantation Recipients: 2017 Update. J. Clin. Oncol..

[12] Cecinati V., Guastadisegni C., Russo F.G., Brescia L.P. (2013). Antifungal therapy in children: An update. Eur. J. Pediatr..

[13] Groll A.H., Tragiannidis A. (2010). Update on antifungal agents for paediatric patients. Clin. Microbiol. Infect..

[14] Pana Z.D., Vikelouda K., Roilides E. (2014). Rare fungal infections in children: An updated review of the literature. Curr. Fungal Infect. Rep..

[15] Soltani M., Tobin C.M., Bowker K.E., Sunderland J., MacGowan A.P., Lovering A.M. (2006). Evidence of excessive concentrations of 5-flucytosine in children aged below 12 years: A 12-year review of serum concentrations from a UK clinical assay reference laboratory. Int. J. Antimicrob. Agents.

[16] Almirante B., Rodriguez D. (2007). Antifungal agents in neonates: Issues and recommendations. Paediatr. Drugs.

[17] Leonart L.P., Tonin F.S., Ferreira V.L., Tavares da Silva Penteado S., de Araujo Motta F., Pontarolo R. (2017). Fluconazole Doses Used for Prophylaxis of Invasive Fungal Infection in Neonatal Intensive Care Units: A Network Meta-Analysis. J. Pediatr..

[18] Dvorak C.C., Fisher B.T., Sung L., Steinbach W.J., Nieder M., Alexander S., Zaoutis T.E. (2012). Antifungal prophylaxis in pediatric hematology/oncology: New choices & new data. Pediatr. Blood Cancer.

[19] Goodman J.L., Winston D.J., Greenfield R.A., Chandrasekar P.H., Fox B., Kaizer H., Shadduck R.K., Shea T.C., Stiff P., Friedman D.J. (1992). A controlled trial of fluconazole to prevent fungal infections in patients undergoing bone marrow transplantation. N. Engl. J. Med..

[20] Slavin M.A., Osborne B., Adams R., Levenstein M.J., Schoch H.G., Feldman A.R., Meyers J.D., Bowden R.A. (1995). Efficacy and safety of fluconazole prophylaxis for fungal infections after marrow transplantation—A prospective, randomized, double-blind study. J. Infect. Dis..

[21] Marr K.A., Seidel K., Slavin M.A., Bowden R.A., Schoch H.G., Flowers M.E., Corey L., Boeckh M. (2000). Prolonged fluconazole prophylaxis is associated with persistent protection against candidiasis-related death in allogeneic marrow transplant recipients: Long-term follow-up of a randomized, placebo-controlled trial. Blood.

[22] Pappas P.G., Kauffman C.A., Andes D., Benjamin D.K., Calandra T.F., Edwards J.E., Filler S.G., Fisher J.F., Kullberg B.J., Ostrosky-Zeichner L. (2009). Clinical practice guidelines for the management of candidiasis: 2009 update by the Infectious Diseases Society of America. Clin. Infect. Dis..

[23] Science M., Robinson P.D., MacDonald T., Rassekh S.R., Dupuis L.L., Sung L. (2014). Guideline for primary antifungal prophylaxis for pediatric patients with cancer or hematopoietic stem cell transplant recipients. Pediatr. Blood Cancer.

[24] Novelli V., Holzel H. (1999). Safety and tolerability of fluconazole in children. Antimicrob. Agents Chemother..

[25] Aguilar C., Malphettes M., Donadieu J., Chandesris O., Coignard-Biehler H., Catherinot E., Pellier I., Stephan J.L., Le Moing V., Barlogis V. (2014). Prevention of infections during primary immunodeficiency. Clin. Infect. Dis..

[26] Antachopoulos C. (2010). Invasive fungal infections in congenital immunodeficiencies. Clin. Microbiol. Infect..

[27] Ullmann A.J., Aguado J.M., Arikan-Akdagli S., Denning D.W., Groll A.H., Lagrou K., Lass-Florl C., Lewis R.E., Munoz P., Verweij P.E. (2018). Diagnosis and management of Aspergillus diseases: Executive summary of the 2017 ESCMID-ECMM-ERS guideline. Clin. Microbiol. Infect..

[28] Mead L., Danziger-Isakov L.A., Michaels M.G., Goldfarb S., Glanville A.R., Benden C., International Pediatric Lung Transplant Collaborative (2014). Antifungal prophylaxis in pediatric lung transplantation: An international multicenter survey. Pediatr. Transplant..

[29] Vardakas K.Z., Michalopoulos A., Falagas M.E. (2005). Fluconazole versus itraconazole for antifungal prophylaxis in neutropenic patients with haematological malignancies: A meta-analysis of randomised-controlled trials. Br. J. Haematol..

[30] Marr K.A., Crippa F., Leisenring W., Hoyle M., Boeckh M., Balajee S.A., Nichols W.G., Musher B., Corey L. (2004). Itraconazole versus fluconazole for prevention of fungal infections in patients receiving allogeneic stem cell transplants. Blood.

[31] Wingard J.R., Carter S.L., Walsh T.J., Kurtzberg J., Small T.N., Baden L.R., Gersten I.D., Mendizabal A.M., Leather H.L., Confer D.L. (2010). Randomized, double-blind trial of fluconazole versus voriconazole for prevention of invasive fungal infection after allogeneic hematopoietic cell transplantation. Blood.

[32] Zembles T.N., Thompson N.E., Havens P.L., Kaufman B.A., Huppler A.R. (2016). An Optimized Voriconazole Dosing Strategy to Achieve Therapeutic Serum Concentrations in Children Younger than 2 Years Old. Pharmacotherapy.

[33] Walsh T.J., Lutsar I., Driscoll T., Dupont B., Roden M., Ghahramani P., Hodges M., Groll A.H., Perfect J.R. (2002). Voriconazole in the treatment of aspergillosis, scedosporiosis and other invasive fungal infections in children. Pediatr. Infect. Dis. J..

[34] Patterson T.F., Thompson G.R., Denning D.W., Fishman J.A., Hadley S., Herbrecht R., Kontoyiannis D.P., Marr K.A., Morrison V.A., Nguyen M.H. (2016). Practice Guidelines for the Diagnosis and Management of Aspergillosis: 2016 Update by the Infectious Diseases Society of America. Clin. Infect. Dis..

[35] Cesaro S., Milano G.M., Aversa F. (2011). Retrospective survey on the off-label use of posaconazole in pediatric hematology patients. Eur. J. Clin. Microbiol. Infect. Dis..

[36] Jancel T., Shaw P.A., Hallahan C.W., Kim T., Freeman A.F., Holland S.M., Penzak S.R. (2017). Therapeutic drug monitoring of posaconazole oral suspension in paediatric patients younger than 13 years of age: A retrospective analysis and literature review. J. Clin. Pharm. Ther..

[37] Vanstraelen K., Colita A., Bica A.M., Mols R., Augustijns P., Peersman N., Vermeersch P., Annaert P., Spriet I. (2016). Pharmacokinetics of Posaconazole Oral Suspension in Children Dosed According to Body Surface Area. Pediatr Infect. Dis. J..

[38] Doring M., Cabanillas Stanchi K.M., Queudeville M., Feucht J., Blaeschke F., Schlegel P., Feuchtinger T., Lang P., Muller I., Handgretinger R. (2017). Efficacy, safety and feasibility of antifungal prophylaxis with posaconazole tablet in paediatric patients after haematopoietic stem cell transplantation. J. Cancer Res. Clin. Oncol..

[39] Vicenzi E.B., Cesaro S. (2018). Posaconazole in immunocompromised pediatric patients. Expert. Rev. Anti Infect. Ther..

[40] Scott L.J. (2017). Micafungin: A Review in the Prophylaxis and Treatment of Invasive Candida Infections in Paediatric Patients. Paediatr. Drugs.

[41] Manzoni P., Wu C., Tweddle L., Roilides E. (2014). Micafungin in Premature and Non-Premature Infants: A Systematic Review of Nine Clinical Trials. Pediatr. Infect. Dis. J..

[42] Ostrosky-Zeichner L., Kontoyiannis D., Raffalli J., Mullane K.M., Vazquez J., Anaissie E.J., Lipton J., Jacobs P., van Rensburg J.H., Rex J.H. (2005). International, open-label, noncomparative, clinical trial of micafungin alone and in combination for treatment of newly diagnosed and refractory candidemia. Eur. J. Clin. Microbiol. Infect. Dis..

[43] Wasmann R.E., Muilwijk E.W., Burger D.M., Verweij P.E., Knibbe C.A., Bruggemann R.J. (2018). Clinical Pharmacokinetics and Pharmacodynamics of Micafungin. Clin. Pharmacokinet..

[44] Hope W.W., Mickiene D., Petraitis V., Petraitiene R., Kelaher A.M., Hughes J.E., Cotton M.P., Bacher J., Keirns J.J., Buell D. (2008). The pharmacokinetics and pharmacodynamics of micafungin in experimental hematogenous Candida meningoencephalitis: Implications for echinocandin therapy in neonates. J. Infect. Dis..

[45] Hope W.W., Smith P.B., Arrieta A., Buell D.N., Roy M., Kaibara A., Walsh T.J., Cohen-Wolkowiez M., Benjamin D.K. (2010). Population pharmacokinetics of micafungin in neonates and young infants. Antimicrob. Agents Chemother..

[46] Leroux S., Jacqz-Aigrain E., Elie V., Legrand F., Barin-Le Guellec C., Aurich B., Biran V., Dusang B., Goudjil S., Coopman S. (2018). Pharmacokinetics and safety of fluconazole and micafungin in neonates with systemic candidiasis: A randomized, open-label clinical trial. Br. J. Clin. Pharmacol..

[47] Auriti C., Piersigilli F., Ronchetti M.P., Campi F., Amante P.G., Falcone M., Goffredo B.M. (2016). Shunt lock therapy with micafungin to treat shunt-associated Candida albicans meningitis in an infant. J. Antimicrob. Chemother..

[48] Van Burik J.A., Ratanatharathorn V., Stepan D.E., Miller C.B., Lipton J.H., Vesole D.H., Bunin N., Wall D.A., Hiemenz J.W., Satoi Y. (2004). Micafungin versus fluconazole for prophylaxis against invasive fungal infections during neutropenia in patients undergoing hematopoietic stem cell transplantation. Clin. Infect. Dis..

[49] Yoshikawa K., Nakazawa Y., Katsuyama Y., Hirabayashi K., Saito S., Shigemura T., Tanaka M., Yanagisawa R., Sakashita K., Koike K. (2014). Safety, tolerability, and feasibility of antifungal prophylaxis with micafungin at 2 mg/kg daily in pediatric patients undergoing allogeneic hematopoietic stem cell transplantation. Infection.

[50] Kobayashi R., Suzuki N., Yoshida M., Iizuka S., Suzuki D., Sano H., Kudoh T. (2013). Efficacy and safety of micafungin for febrile neutropenia in pediatric patients with hematological malignancies: A multicenter prospective study. J. Pediatr. Hematol. Oncol..

[51] Somer A., Torun S.H., Salman N. (2011). Caspofungin therapy in immunocompromised children and neonates. Expert Rev. Anti Infect. Ther..

[52] Jeon G.W., Sin J.B. (2014). Successful caspofungin treatment of persistent candidemia in extreme prematurity at 23 and 24 weeks’ gestation. Taiwan Yi Zhi.

[53] Saez-Llorens X., Macias M., Maiya P., Pineros J., Jafri H.S., Chatterjee A., Ruiz G., Raghavan J., Bradshaw S.K., Kartsonis N.A. (2009). Pharmacokinetics and safety of caspofungin in neonates and infants less than 3 months of age. Antimicrob. Agents Chemother..

[54] Mattiuzzi G.N., Alvarado G., Giles F.J., Ostrosky-Zeichner L., Cortes J., O’Brien S., Verstovsek S., Faderl S., Zhou X., Raad I.I. (2006). Open-label, randomized comparison of itraconazole versus caspofungin for prophylaxis in patients with hematologic malignancies. Antimicrob. Agents Chemother..

[55] Doring M., Hartmann U., Erbacher A., Lang P., Handgretinger R., Muller I. (2012). Caspofungin as antifungal prophylaxis in pediatric patients undergoing allogeneic hematopoietic stem cell transplantation: A retrospective analysis. BMC Infect. Dis..

[56] Maximova N., Schillani G., Simeone R., Maestro A., Zanon D. (2017). Comparison of Efficacy and Safety of Caspofungin Versus Micafungin in Pediatric Allogeneic Stem Cell Transplant Recipients: A Retrospective Analysis. Adv. Ther..

[57] Kumar S., Bansal A., Chakrabarti A., Singhi S. (2013). Evaluation of efficacy of probiotics in prevention of candida colonization in a PICU-a randomized controlled trial. Crit. Care Med..

[58] Kumar S., Singhi S. (2013). Role of probiotics in prevention of Candida infection in critically ill children. Mycoses.

[59] Manzoni P., Stolfi I., Messner H., Cattani S., Laforgia N., Romeo M.G., Bollani L., Rinaldi M., Gallo E., Quercia M. (2012). Bovine lactoferrin prevents invasive fungal infections in very low birth weight infants: A randomized controlled trial. Pediatrics.

[60] Demirel G., Celik I.H., Erdeve O., Saygan S., Dilmen U., Canpolat F.E. (2013). Prophylactic Saccharomyces boulardii versus nystatin for the prevention of fungal colonization and invasive fungal infection in premature infants. Eur. J. Pediatr..

[61] Srinivasjois R., Rao S., Patole S. (2013). Prebiotic supplementation in preterm neonates: Updated systematic review and meta-analysis of randomised controlled trials. Clin. Nutr..

[62] Hundalani S., Pammi M. (2013). Invasive fungal infections in newborns and current management strategies. Expert Rev. Anti Infect. Ther..

[63] Tiffany K.F., Smith P.B., Benjamin D.K. (2005). Neonatal candidiasis: Prophylaxis and treatment. Expert Opin. Pharmacother..

[64] Kaufman D.A. (2010). Challenging issues in neonatal candidiasis. Curr. Med. Res. Opin..

[65] Kaufman D., Boyle R., Hazen K.C., Patrie J.T., Robinson M., Donowitz L.G. (2001). Fluconazole prophylaxis against fungal colonization and infection in preterm infants. N. Engl. J. Med..

[66] Manzoni P., Jacqz-Aigrain E., Rizzollo S., Franco C., Stronati M., Mostert M., Farina D. (2011). Antifungal prophylaxis in neonates. Early Hum. Dev..

[67] Healy C.M., Campbell J.R., Zaccaria E., Baker C.J. (2008). Fluconazole prophylaxis in extremely low birth weight neonates reduces invasive candidiasis mortality rates without emergence of fluconazole-resistant Candida species. Pediatrics.

[68] Benjamin D.K., Hudak M.L., Duara S., Randolph D.A., Bidegain M., Mundakel G.T., Natarajan G., Burchfield D.J., White R.D., Shattuck K.E. (2014). Effect of fluconazole prophylaxis on candidiasis and mortality in premature infants: A randomized clinical trial. JAMA.

[69] Ericson J.E., Kaufman D.A., Kicklighter S.D., Bhatia J., Testoni D., Gao J., Smith P.B., Prather K.O., Benjamin D.K., Fluconazole Prophylaxis Study Team on behalf of the Best Pharmaceuticals for Children Act-Pediatric Trials Network Steering Committee (2016). Fluconazole Prophylaxis for the Prevention of Candidiasis in Premature Infants: A Meta-analysis Using Patient-level Data. Clin. Infect. Dis..

[70] Kaufman D.A., Cuff A.L., Wamstad J.B., Boyle R., Gurka M.J., Grossman L.B., Patrick P. (2011). Fluconazole prophylaxis in extremely low birth weight infants and neurodevelopmental outcomes and quality of life at 8 to 10 years of age. J. Pediatr..

[71] Aydemir C., Oguz S.S., Dizdar E.A., Akar M., Sarikabadayi Y.U., Saygan S., Erdeve O., Dilmen U. (2011). Randomised controlled trial of prophylactic fluconazole versus nystatin for the prevention of fungal colonisation and invasive fungal infection in very low birth weight infants. Arch. Dis. Child. Fetal Neonatal Ed..

[72] Violaris K., Carbone T., Bateman D., Olawepo O., Doraiswamy B., LaCorte M. (2010). Comparison of fluconazole and nystatin oral suspensions for prophylaxis of systemic fungal infection in very low birthweight infants. Am. J. Perinatol..

[73] Austin N., Darlow B.A., McGuire W. (2013). Prophylactic oral/topical non-absorbed antifungal agents to prevent invasive fungal infection in very low birth weight infants. Cochrane Database Syst. Rev..

[74] Lestner J.M., Smith P.B., Cohen-Wolkowiez M., Benjamin D.K., Hope W.W. (2013). Antifungal agents and therapy for infants and children with invasive fungal infections: A pharmacological perspective. Br. J. Clin. Pharmacol..

[75] Fortmann I., Hartz A., Paul P., Pulzer F., Muller A., Bottger R., Proquitte H., Dawczynski K., Simon A., Rupp J. (2018). Antifungal Treatment and Outcome in Very-Low-Birth-Weight-Infants—A Population-Based Observational Study of the German Neonatal Network. Pediatr. Infect. Dis. J..

[76] Greenberg R.G., Benjamin D.K. (2014). Neonatal candidiasis: Diagnosis, prevention, and treatment. J. Infect..

[77] Greenberg R.G., Benjamin D.K., Gantz M.G., Cotten C.M., Stoll B.J., Walsh M.C., Sanchez P.J., Shankaran S., Das A., Higgins R.D. (2012). Empiric antifungal therapy and outcomes in extremely low birth weight infants with invasive candidiasis. J. Pediatr..

[78] Levy I., Shalit I., Birk E., Sirota L., Ashkenazi S., German B., Linder N. (2006). Candida endocarditis in neonates: Report of five cases and review of the literature. Mycoses.

[79] Calitri C., Caviglia I., Cangemi G., Furfaro E., Bandettini R., Fioredda F., Amoroso L., Faraci M., Risso F.M., Mattioli G. (2017). Performance of 1,3-β-d-glucan for diagnosing invasive fungal diseases in children. Mycoses.

[80] Goudjil S., Kongolo G., Dusol L., Imestouren F., Cornu M., Leke A., Chouaki T. (2013). (1-3)-β-d-glucan levels in candidiasis infections in the critically ill neonate. J. Matern. Fetal Med..

[81] Cornu M., Goudjil S., Kongolo G., Leke A., Poulain D., Chouaki T., Sendid B. (2018). Evaluation of the (1,3)-β-d-glucan assay for the diagnosis of neonatal invasive yeast infections. Med. Mycol..

[82] Ramos J.T., Villar S., Bouza E., Bergon-Sendin E., Perez Rivilla A., Collados C.T., Andreu M., Reyes C.S., Campos-Herrero M.I., de Heredia J.L. (2017). Performance of a Quantitative PCR-Based Assay and β-d-Glucan Detection for Diagnosis of Invasive Candidiasis in Very-Low-Birth-Weight Preterm Neonatal Patients (CANDINEO Study). J. Clin. Microbiol..

[83] Hamula C.L., Hughes K., Fisher B.T., Zaoutis T.E., Singh I.R., Velegraki A. (2016). T2Candida Provides Rapid and Accurate Species Identification in Pediatric Cases of Candidemia. Am. J. Clin. Pathol..

[84] Baley J.E., Meyers C., Kliegman R.M., Jacobs M.R., Blumer J.L. (1990). Pharmacokinetics, outcome of treatment, and toxic effects of amphotericin B and 5-fluorocytosine in neonates. J. Pediatr..

[85] Lopez Sastre J.B., Coto Cotallo G.D., Fernandez Colomer B. (2003). Neonatal invasive candidiasis: A prospective multicenter study of 118 cases. Am. J. Perinatol..

[86] Cetin H., Yalaz M., Akisu M., Hilmioglu S., Metin D., Kultursay N. (2005). The efficacy of two different lipid-based amphotericin B in neonatal Candida septicemia. Pediatr. Int..

[87] Jeon G.W., Koo S.H., Lee J.H., Hwang J.H., Kim S.S., Lee E.K., Chang W., Chang Y.S., Park W.S. (2007). A comparison of AmBisome to amphotericin B for treatment of systemic candidiasis in very low birth weight infants. Yonsei Med. J..

[88] Pfaller M.A., Rhomberg P.R., Messer S.A., Jones R.N., Castanheira M. (2015). Isavuconazole, micafungin, and 8 comparator antifungal agents’ susceptibility profiles for common and uncommon opportunistic fungi collected in 2013: Temporal analysis of antifungal drug resistance using CLSI species-specific clinical breakpoints and proposed epidemiological cutoff values. Diagn. Microbiol. Infect. Dis..

[89] Berkow E.L., Lockhart S.R. (2017). Fluconazole resistance in Candida species: A current perspective. Infect. Drug Resist..

[90] Benedict K., Roy M., Kabbani S., Anderson E.J., Farley M.M., Harb S., Harrison L.H., Bonner L., Wadu V.L., Marceaux K. (2018). Neonatal and Pediatric Candidemia: Results From Population-Based Active Laboratory Surveillance in Four US Locations, 2009–2015. J. Pediatr. Infect. Dis. Soc..

[91] Caggiano G., Lovero G., De Giglio O., Barbuti G., Montagna O., Laforgia N., Montagna M.T. (2017). Candidemia in the Neonatal Intensive Care Unit: A Retrospective, Observational Survey and Analysis of Literature Data. BioMed Res. Int..

[92] Warris A., Pana Z.D., Oletto A.R.L., Roilides E., EUROCANDY Study Group Antifungal drug susceptibility of *candida* spp. in neonatal and paediatric candidaemia: A european multi-centre retrospective study (EUROCANDY). Proceedings of the 36th Annual Meeting of The European Society For Pediatric Infectious Diseases ESPID 2018.

[93] Benjamin D.K., Kaufman D.A., Hope W.W., Smith P.B., Arrieta A., Manzoni P., Kovanda L.L., Lademacher C., Isaacson B., Jednachowski D. (2018). A Phase 3 Study of Micafungin Versus Amphotericin B Deoxycholate in Infants With Invasive Candidiasis. Pediatr. Infect. Dis. J..

[94] Auriti C., Falcone M., Ronchetti M.P., Goffredo B.M., Cairoli S., Crisafulli R., Piersigilli F., Corsetti T., Dotta A., Pai M.P. (2016). High-Dose Micafungin for Preterm Neonates and Infants with Invasive and Central Nervous System Candidiasis. Antimicrob. Agents Chemother..

[95] Caudle K.E., Inger A.G., Butler D.R., Rogers P.D. (2012). Echinocandin use in the neonatal intensive care unit. Ann. Pharmacother..

[96] Jans J., Bruggemann R.J., Christmann V., Verweij P.E., Warris A. (2013). Favorable outcome of neonatal cerebrospinal fluid shunt-associated Candida meningitis with caspofungin. Antimicrob. Agents Chemother..

[97] Warn P.A., Livermore J., Howard S., Felton T.W., Sharp A., Gregson L., Goodwin J., Petraitiene R., Petraitis V., Cohen-Wolkowiez M. (2012). Anidulafungin for neonatal hematogenous Candida meningoencephalitis: Identification of candidate regimens for humans using a translational pharmacological approach. Antimicrob. Agents Chemother..

[98] Wilke M.H. (2013). Invasive fungal infections in infants-focus on anidulafungin. Clin. Med. Insights Pediatr..

[99] Cohen-Wolkowiez M., Benjamin D.K., Piper L., Cheifetz I.M., Moran C., Liu P., Aram J., Kashuba A.D., Capparelli E., Walsh T.J. (2011). Safety and pharmacokinetics of multiple-dose anidulafungin in infants and neonates. Clin. Pharmacol. Ther..

[100] Ethier M.C., Science M., Beyene J., Briel M., Lehrnbecher T., Sung L. (2012). Mould-active compared with fluconazole prophylaxis to prevent invasive fungal diseases in cancer patients receiving chemotherapy or haematopoietic stem-cell transplantation: A systematic review and meta-analysis of randomised controlled trials. Br. J. Cancer.

[101] Yu D.T., Peterson J.F., Seger D.L., Gerth W.C., Bates D.W. (2005). Frequency of potential azole drug-drug interactions and consequences of potential fluconazole drug interactions. Pharmacoepidemiol. Drug Saf..

[102] Gastine S., Lehrnbecher T., Muller C., Farowski F., Bader P., Ullmann-Moskovits J., Cornely O.A., Groll A.H., Hempel G. (2018). Pharmacokinetic Modeling of Voriconazole to Develop an Alternative Dosing Regimen in Children. Antimicrob. Agents Chemother..

[103] Pana Z.D., Roilides E. (2011). Risk of azole-enhanced vincristine neurotoxicity in pediatric patients with hematological malignancies: Old problem—new dilemma. Pediatr. Blood Cancer.

[104] Pana Z.D., Kourti M., Vikelouda K., Vlahou A., Katzilakis N., Papageorgiou M., Doganis D., Petrikkos L., Paisiou A., Koliouskas D. (2018). Voriconazole Antifungal Prophylaxis in Children With Malignancies: A Nationwide Study. J. Pediatr. Hematol. Oncol..

[105] Allegra S., Fatiguso G., De Francia S., Favata F., Pirro E., Carcieri C., De Nicolo A., Cusato J., Di Perri G., D’Avolio A. (2018). Therapeutic drug monitoring of voriconazole for treatment and prophylaxis of invasive fungal infection in children. Br. J. Clin. Pharmacol..

[106] Ullmann A.J., Lipton J.H., Vesole D.H., Chandrasekar P., Langston A., Tarantolo S.R., Greinix H., Morais de Azevedo W., Reddy V., Boparai N. (2007). Posaconazole or fluconazole for prophylaxis in severe graft-versus-host disease. N. Engl. J. Med..

[107] Doring M., Muller C., Johann P.D., Erbacher A., Kimmig A., Schwarze C.P., Lang P., Handgretinger R., Muller I. (2012). Analysis of posaconazole as oral antifungal prophylaxis in pediatric patients under 12 years of age following allogeneic stem cell transplantation. BMC Infect. Dis..

[108] Doring M., Blume O., Haufe S., Hartmann U., Kimmig A., Schwarze C.P., Lang P., Handgretinger R., Muller I. (2014). Comparison of itraconazole, voriconazole, and posaconazole as oral antifungal prophylaxis in pediatric patients following allogeneic hematopoietic stem cell transplantation. Eur. J. Clin. Microbiol. Infect. Dis..

[109] Doring M., Eikemeier M., Cabanillas Stanchi K.M., Hartmann U., Ebinger M., Schwarze C.P., Schulz A., Handgretinger R., Muller I. (2015). Antifungal prophylaxis with posaconazole vs. fluconazole or itraconazole in pediatric patients with neutropenia. Eur. J. Clin. Microbiol. Infect. Dis..

[110] Verweij P.E., Warris A., Weemaes C.M. (2003). Preventing fungal infections in chronic granulomatous disease. N. Engl. J. Med..

[111] Welzen M.E., Bruggemann R.J., Van Den Berg J.M., Voogt H.W., Gilissen J.H., Pajkrt D., Klein N., Burger D.M., Warris A. (2011). A twice daily posaconazole dosing algorithm for children with chronic granulomatous disease. Pediatr. Infect. Dis. J..

[112] Morgan J.E., Hassan H., Cockle J.V., Lethaby C., James B., Phillips R.S. (2017). Critical review of current clinical practice guidelines for antifungal therapy in paediatric haematology and oncology. Support. Care Cancer.

[113] Santolaya M.E., Alvarez A.M., Acuna M., Aviles C.L., Salgado C., Tordecilla J., Varas M., Venegas M., Villarroel M., Zubieta M. (2018). Efficacy of pre-emptive versus empirical antifungal therapy in children with cancer and high-risk febrile neutropenia: A randomized clinical trial. J. Antimicrob. Chemother..

[114] Maertens J.A., Madero L., Reilly A.F., Lehrnbecher T., Groll A.H., Jafri H.S., Green M., Nania J.J., Bourque M.R., Wise B.A. (2010). A randomized, double-blind, multicenter study of caspofungin versus liposomal amphotericin B for empiric antifungal therapy in pediatric patients with persistent fever and neutropenia. Pediatr. Infect. Dis. J..

[115] Cortegiani A., Russotto V., Maggiore A., Attanasio M., Naro A.R., Raineri S.M., Giarratano A. (2016). Antifungal agents for preventing fungal infections in non-neutropenic critically ill patients. Cochrane Database Syst. Rev..

[116] Huppler A.R., Fisher B.T., Lehrnbecher T., Walsh T.J., Steinbach W.J. (2017). Role of Molecular Biomarkers in the Diagnosis of Invasive Fungal Diseases in Children. J. Pediatr. Infect. Dis. Soc..

[117] Lehrnbecher T., Robinson P.D., Fisher B.T., Castagnola E., Groll A.H., Steinbach W.J., Zaoutis T.E., Negeri Z.F., Beyene J., Phillips B. (2016). Galactomannan, β-d-Glucan, and Polymerase Chain Reaction-Based Assays for the Diagnosis of Invasive Fungal Disease in Pediatric Cancer and Hematopoietic Stem Cell Transplantation: A Systematic Review and Meta-Analysis. Clin. Infect. Dis..

[118] Gupta P., Ahmad A., Khare V., Kumar A., Banerjee G., Verma N., Singh M. (2017). Comparative evaluation of pan-fungal real-time PCR, galactomannan and (1-3)-β-d-glucan assay for invasive fungal infection in paediatric cancer patients. Mycoses.

[119] Zacharioudakis I.M., Zervou F.N., Mylonakis E. (2018). Use of T2MR in invasive candidiasis with and without candidemia. Future Microbiol..

[120] Lestner J.M., Versporten A., Doerholt K., Warris A., Roilides E., Sharland M., Bielicki J., Goossens H., Group A.P. (2015). Systemic antifungal prescribing in neonates and children: Outcomes from the Antibiotic Resistance and Prescribing in European Children (ARPEC) Study. Antimicrob. Agents Chemother..

[121] Zaoutis T.E., Jafri H.S., Huang L.M., Locatelli F., Barzilai A., Ebell W., Steinbach W.J., Bradley J., Lieberman J.M., Hsiao C.C. (2009). A prospective, multicenter study of caspofungin for the treatment of documented Candida or Aspergillus infections in pediatric patients. Pediatrics.

[122] Maertens J., Raad I., Petrikkos G., Boogaerts M., Selleslag D., Petersen F.B., Sable C.A., Kartsonis N.A., Ngai A., Taylor A. (2004). Efficacy and safety of caspofungin for treatment of invasive aspergillosis in patients refractory to or intolerant of conventional antifungal therapy. Clin. Infect. Dis..

[123] Rosanova M.T., Bes D., Serrano Aguilar P., Cuellar Pompa L., Sberna N., Lede R. (2016). Efficacy and safety of caspofungin in children: Systematic review and meta-analysis. Arch. Argent. Pediatr..

[124] Benjamin D.K., Deville J.G., Azie N., Kovanda L., Roy M., Wu C., Arrieta A. (2013). Safety and pharmacokinetic profiles of repeated-dose micafungin in children and adolescents treated for invasive candidiasis. Pediatr. Infect. Dis. J..

[125] Queiroz-Telles F., Berezin E., Leverger G., Freire A., van der Vyver A., Chotpitayasunondh T., Konja J., Diekmann-Berndt H., Koblinger S., Groll A.H. (2008). Micafungin versus liposomal amphotericin B for pediatric patients with invasive candidiasis: Substudy of a randomized double-blind trial. Pediatr. Infect. Dis. J..

[126] Lee C.H., Lin J.C., Ho C.L., Sun M., Yen W.T., Lin C. (2017). Efficacy and safety of micafungin versus extensive azoles in the prevention and treatment of invasive fungal infections for neutropenia patients with hematological malignancies: A meta-analysis of randomized controlled trials. PLoS ONE.

[127] Tsekoura M., Ioannidou M., Pana Z.D., Haidich A.B., Antachopoulos C., Iosifidis E., Kolios G., Roilides E. (2018). Efficacy and Safety of Echinocandins for the Treatment of Invasive Candidiasis in Children: A Meta-Analysis. Pediatr. Infect. Dis. J..

[128] Benjamin D.K., Driscoll T., Seibel N.L., Gonzalez C.E., Roden M.M., Kilaru R., Clark K., Dowell J.A., Schranz J., Walsh T.J. (2006). Safety and pharmacokinetics of intravenous anidulafungin in children with neutropenia at high risk for invasive fungal infections. Antimicrob. Agents Chemother..

[129] Rosanova M.T., Sarkis C., Escarra F., Epelbaum C., Sberna N., Carnovale S., Figueroa C., Bologna R., Lede R. (2017). Anidulafungin in children: Experience in a tertiary care children’s hospital in Argentina. Arch. Argent. Pediatr..

[130] Roilides E., Carlesse F., Leister-Tebbe H., Conte U., Yan J.L., Liu P., Tawadrous M., Aram J.A., Queiroz-Telles F., on behalf of the anidulafungin A8851008 pediatric study group (2018). A Prospective, Open-label Study to Assess the Safety, Tolerability, and Efficacy of Anidulafungin in the Treatment of Invasive Candidiasis in Children Aged 2 to <18 Years Old. Pediatr. Infect. Dis. J..

[131] Pana Z.D., Seidel D., Skiada A., Groll A.H., Petrikkos G., Cornely O.A., Roilides E., Collaborators of Zygomyco.net and/or FungiScope Registries (2016). Invasive mucormycosis in children: An epidemiologic study in European and non-European countries based on two registries. BMC Infect. Dis..

[132] Lehrnbecher T., Attarbaschi A., Duerken M., Garbino J., Gruhn B., Kontny U., Luer S., Phillips R., Scholz J., Wagner H.J. (2010). Posaconazole salvage treatment in paediatric patients: A multicentre survey. Eur. J. Clin. Microbiol. Infect. Dis..

[133] Vehreschild J.J., Birtel A., Vehreschild M.J., Liss B., Farowski F., Kochanek M., Sieniawski M., Steinbach A., Wahlers K., Fatkenheuer G. (2013). Mucormycosis treated with posaconazole: Review of 96 case reports. Crit. Rev. Microbiol..

[134] Cornely O.A., Arikan-Akdagli S., Dannaoui E., Groll A.H., Lagrou K., Chakrabarti A., Lanternier F., Pagano L., Skiada A., Akova M. (2013). ESCMID and ECMM joint clinical guidelines for the diagnosis and management of mucormycosis 2013. Clin. Microbiol. Infect..

[135] Krishna G., Sansone-Parsons A., Martinho M., Kantesaria B., Pedicone L. (2007). Posaconazole plasma concentrations in juvenile patients with invasive fungal infection. Antimicrob. Agents Chemother..

[136] Vicenzi E.B., Calore E., Decembrino N., Berger M., Perruccio K., Carraro F., Rossin S., Putti M.C., Molinaro M., Tridello G. (2018). Posaconazole oral dose and plasma levels in pediatric hematology-oncology patients. Eur. J. Haematol..

[137] Miceli M.H., Kauffman C.A. (2015). Isavuconazole: A New Broad-Spectrum Triazole Antifungal Agent. Clin. Infect. Dis..

[138] Barg A.A., Malkiel S., Bartuv M., Greenberg G., Toren A., Keller N. (2018). Successful treatment of invasive mucormycosis with isavuconazole in pediatric patients. Pediatr. Blood Cancer.

[139] Pana Z.D., Vikelouda K., Roilides E. (2014). Rare Fungal Infections in Children: An Updated Review of the Literature. Curr. Fungal Infect. Rep..

[140] Katragkou A., Roilides E. (2012). Immunotherapy of infections caused by rare filamentous fungi. Clin. Microbiol. Infect..

